# Vitamin D status and its determinants in German elite athletes

**DOI:** 10.1007/s00421-024-05699-6

**Published:** 2025-01-04

**Authors:** Sebastian Hacker, Claudia Lenz, Lukas Reichert, Robert Ringseis, Karen Zentgraf, Karsten Krüger

**Affiliations:** 1 Department of Exercise Physiology and Sports Therapy, Institute of Sports Science, Justus Liebig University Giessen, Kugelberg 62, 35394 Giessen, Germany; 2 Work Unit Movement and Exercise Science in Sports, Institute of Sport Sciences, Goethe University Frankfurt, Ginnheimer Landstraße 39, 60487 Frankfurt am Main, Germany; 3 Institute of Animal Nutrition and Nutrition Physiology, Justus Liebig University Giessen, Heinrich-Buff-Ring 26-32, 35392 Giessen, Germany

**Keywords:** Vitamin D, 25-Hydroxyvitamin D, *VDBP*, SNPs, Elite athletes

## Abstract

**Purpose:**

This study investigated elite German athletes to (1) assess their serum 25(OH)D levels and the prevalence of insufficiency, (2) identify key factors influencing serum 25(OH)D levels, and (3) analyze the association between serum 25(OH)D levels and handgrip strength.

**Methods:**

In this cross-sectional study, a total of 474 athletes (231 female), aged 13–39 years (mean 19.3 years), from ten Olympic disciplines were included. Serum 25(OH)D levels were analyzed via liquid chromatography–mass spectrometry, and 17 single nucleotide polymorphisms (SNPs) related to vitamin D metabolism were determined using leukocyte DNA. Grip strength was measured unimanually using a hand-held dynamometer. Multiple linear regressions were used to analyze the influence of SNPs, age, sex, season (summer vs. winter), and discipline (indoor vs. outdoor) on 25(OH)D status. Linear regression analyzed the relationship between handgrip strength and serum 25(OH)D levels.

**Results:**

In total, 55.5% (*n* = 263) of athletes demonstrated insufficient serum 25(OH)D levels (< 30 ng/mL, < 75 nmol/L), with 16% (*n* = 76) showing levels below 20 ng/mL (50 nmol/L). After correction for multiple testing, significant influences on 25(OH)D levels were observed for the C allele of *VDBP* rs7041 (AC Genotype: $\widehat{\upbeta }$ = 7.46, *p* < .001; CC Genotype: $\widehat{\upbeta }$ = 6.23, *p* = .001). Age (all *p* < .01) and discipline (indoor vs. outdoor; all *p* < .05) also influenced serum 25(OH)D levels. Furthermore, serum 25(OH)D was positively associated with handgrip strength ($\widehat{\upbeta }$= 0.01, *p* < .001).

**Conclusion:**

A high prevalence of insufficient 25(OH)D levels (< 30 ng/mL, < 75 nmol/L) was observed. We identified certain genetic variants as well as age and discipline as predictors of serum 25(OH)D levels. This knowledge may guide individualized diagnostic, nutritional, and supplementation strategies.

**Supplementary Information:**

The online version contains supplementary material available at 10.1007/s00421-024-05699-6.

## Introduction

Vitamin D is an essential secosteroid hormone that plays a pivotal role in maintaining overall health, particularly regarding bone health, immune function, and muscle performance (Abrams et al. 2018; Harju et al. 2022). Low vitamin D levels have been associated with an increased risk of stress fractures, respiratory tract infections, and muscle injuries (Crescioli 2022; de la Puente Yagüe et al. 2020), highlighting the importance of adequate vitamin D levels for elite athletes, as these factors contribute to a greater risk of time lost to illness and/or injury (Bauer et al. 2018; Harju et al. 2022). However, different organizations, such as the Institute of Medicine (IOM) and the Endocrine Society, use varying thresholds to define vitamin D status (Holick et al. 2011; Institute of Medicine 2011), complicating the standardization of reporting vitamin D insufficiency (de la Puente Yagüe et al. 2020; Holick 2007; Vieth and Holick 2018). This inconsistency is further compounded by variability in population characteristics, geographical location (latitude), and analytical methods (Cui et al. 2023; Harju et al. 2022). In accordance with guidelines emphasizing the pleiotropic effects of vitamin D (Holick et al. 2011; Pludowski et al. 2018), this study categorizes serum 25-hydroxyvitamin D [25(OH)D] levels < 20 ng/mL (< 50 nmol/L) as ‘deficient’, between 20 ng/mL and 30 ng/mL (50–75 nmol/L) as ‘insufficient’, and ≥ 30 ng/mL as ‘sufficient’ (≥ 75 nmol/L).

Vitamin D exists in two main forms: D2 (ergocalciferol), obtained from the diet, and D3 (cholecalciferol), primarily synthesized in the skin upon exposure to ultraviolet B radiation (Bikle 2014). Both forms are hydroxylated in the liver to form 25-hydroxyvitamin D [25(OH)D] (Bikle 2014), the primary circulating form and biomarker for vitamin D status, reflecting endogenous synthesis (80–90%) and dietary intake (10–20%) (Allison et al. 2015; Owens et al. 2018). Its serum half-life is approximately 3 weeks (Wootton 2005). In the kidneys, 25(OH)D is further hydroxylated to form the biologically active 1,25-hydroxycholecalciferol [1,25(OH)_2_D] (Bikle 2014).

Albeit its vital role in physiological processes, vitamin D insufficiency is widespread, with studies in the US, Canada, and Europe reporting prevalence rates of insufficiency at 24%, 36.8%, and 40.4%, respectively (Cashman 2020). In Germany, 61.5% of adults between 18 and 79 years have 25(OH)D levels below 20 ng/mL (50 nmol/L) (Rabenberg & Mensink 2016). Among elite athletes, a systematic review and meta-analysis by Harju et al. found that 30% (95% confidence interval (CI) 22–39%) of adults and 39% (95% CI 25–55%) of adolescents had 25(OH)D levels below 20 ng/mL (50 nmol/L) (Harju et al. 2022). Another meta-analysis by Farrokhyar et al., using a higher cut-off at 32 ng/mL (80 nmol/L), reported a 56% (95% CI 44–67%) prevalence of insufficiency, with higher risks observed during winter and spring (RR 1.85; 95% CI [1.27, 2.7]), as well as in athletes engaged in indoor sports (RR 1.19; 95% CI [1.09, 1.3]) (Farrokhyar et al. 2015). These findings suggest that factors such as geographical location (latitude), season, discipline, sunscreen use, and skin pigmentation influence vitamin D status (Crescioli 2022), and may thus be helpful when interpreting individual vitamin D levels.

In addition to environmental and lifestyle factors, genetic differences also play a critical role in determining vitamin D levels. Genome-wide association studies (GWAS) estimate the heritability of serum 25(OH)D levels between 13 and 16% (Manousaki et al. 2020; Revez et al. 2020). Single nucleotide polymorphisms (SNPs) within genes related to the vitamin D pathway, such as the vitamin D receptor (*VDR*), vitamin D binding protein (*VDBP*), and enzymes like 7-dehydrocholesterol reductase (*DHCR7*), may affect the conversion, transport, and bioavailability of vitamin D (Bollen et al. 2023; Hyppönen et al. 2022; Krasniqi et al. 2021; Wang et al. 2010). For example, the C allele of the rs7041 polymorphism in the *VDBP* gene has been associated with higher levels of 25(OH)D (Jolliffe et al. 2016; Krasniqi et al. 2021), probably due to the encoded aspartate to glutamate amino acid change, which alters the binding affinity of VDBP for 25(OH)D (Krasniqi et al. 2021). The *DHCR7* gene encodes an enzyme responsible for converting 7-dehydrocholesterol to cholesterol, a precursor of 25(OH)D. Variations in this gene may offer protection against vitamin D deficiency (Wang et al. 2010).

The identification of vitamin D receptors in skeletal muscle tissue (Bischoff et al. 2001; Bischoff-Ferrari et al. 2004) has led to growing evidence suggesting that vitamin D plays a role in muscle function, potentially impacting athletic performance (Abrams et al. 2018; de la Puente Yagüe et al. 2020). One way to assess muscle function is through handgrip strength, a simple and reliable parameter for estimating whole-body strength (Vaidya & Nariya 2021). Grip strength has been shown to predict outcomes, such as physical status and all-cause mortality in various populations (Norman et al. 2011), and is considered a performance-relevant parameter in elite sports (Cronin et al. 2017). However, the association between grip strength and serum 25(OH)D levels remains inconclusive (Książek et al. 2016, 2018; Wium Geiker et al. 2017), warranting further research to derive potential practical implications.

Thus, this study aimed to (1) assess serum 25(OH)D levels and the prevalence of insufficiency, (2) identify key factors influencing serum 25(OH)D levels, including genetic polymorphisms, seasonal variation (summer vs. winter), and discipline (indoor vs. outdoor), and (3) analyze the association between vitamin D levels and handgrip strength. These findings may provide valuable insights to guide personalized diagnostic, nutrition and supplementation strategies. We hypothesized that a substantial proportion of athletes will exhibit insufficient vitamin D levels, and this phenomenon may result from a combination of genetic and environmental factors.

## Materials and methods

This cross-sectional analysis is part of the ongoing “in:prove” project (Individualized performance development in elite sport through holistic and transdisciplinary process optimization) funded by the German Federal Institute of Sport Science (grant number 081901/21–25). In total, 474 elite German athletes (231 female), aged 13–39 years (mean 19.3 years) of ten Olympic disciplines (3 × 3 basketball, artistic gymnastics, bobsleigh, ice hockey, modern pentathlon, rhythmic gymnastics, skeleton, table tennis, trampoline gymnastics, and volleyball), were included. Detailed information regarding the study population is presented in Table 1. To be eligible for this study, the athletes had to be part of a German national squad (junior, perspective or Olympic squad) at the time of measurement. Before the study, all athletes received detailed written and verbal information. All included athletes gave their written informed consent (additionally the athletes’ parents/caretakers in the case of minors). The study protocol was approved by the institutional ethics committee of the University of Giessen, Germany (approval number: AZ 55/22, date of approval: 2022-05-10) and was conducted following the Declaration of Helsinki for human research. STROBE-nut and STROBE-ME reporting guidelines were respected (Gallo et al. 2011; Lachat et al. 2016). Additionally, a participant flowchart can be found in Supplementary Information (SI)1.

**Table 1:** Study population

Variable		*n*	%	Mean ± SD
*Total participants*		474	100	
*Age (years)*				
Over 18		241	50.84	22.4 ± 4.2
Under 18		233	49.16	16.1 ± 1.2
*Sex*				
Female		231	48.73	
Male		243	51.27	
*Season*				
Summer (April–September)		280	59.07	
Winter (October–March)		194	40.93	
*Discipline*				
Indoor		340	71.73	
	Artistic gymnastics	34	10	
	Ice hockey	89	26.18	
	Rhythmic gymnastics	27	7.94	
	Table tennis	17	5	
	Trampoline gymnastics	32	9.41	
	Volleyball	141	41.47	
Outdoor		134	28.27	
	3 × 3 Basketball	45	33.58	
	Bobsleigh	23	17.16	
	Modern pentathlon	35	26.12	
	Skeleton	31	23.13	

*Note.* Disciplines were primarily categorized based on their main place of competition

### Blood sampling

Blood samples for analysis of serum 25(OH)D and genotyping were taken from the median cubital vein of the forearm. On the measurement day, three venous blood samples were collected in 7.5 mL Serum Gel and 7.5 mL and 2.7 mL EDTA tubes (Sarstedt, Nümbrecht, Germany). To prepare for subsequent genotyping, the 7.5 mL EDTA tube was centrifuged at 2000 rpm for 10 min at room temperature, and then the sample was frozen at -80°C. Serum 25(OH)D was analyzed via liquid chromatography–mass spectrometry (LCMS) by an accredited medical laboratory (SYNLAB Medizinisches Versorgungszentrum, Bad Nauheim, Germany). The detection limit was specified in the range of 2–100 ng/mL (5–250 nmol/L).

### Genotyping and SNP selection

DNA was extracted from human whole blood samples using the Chemagic Magnetic Separation Module I (Perkin Elmer Chemagen Technology Inc., Baesweiler, Germany). Genotyping was performed using the Illumina Global Screening Array + Medical Disease + Psych content (GSAv3.0 + MD + Psych) (Illumina Inc, San Diego, CA, USA). All laboratory procedures were conducted according to the manufacturer’s instructions. SNP array raw data was uploaded into, and genotypes were exported from the GenomeStudio2.0 software. The investigated SNPs were chosen based on previous association with the vitamin D pathway (Bollen et al. 2023; Hyppönen et al. 2022; Krasniqi et al. 2021; Wang et al. 2010) and their availability on the aforementioned SNP array. Detailed information regarding the analyzed SNPs can be found in Table 2.

**Table 2:** Gene and single nucleotide polymorphism (SNP) information

Gene	Symbol	Locus	SNP	MAF
Cubilin	*CUBN*	10p13	rs3740165 (T > C)	0.25
Cytochrome P450 24A1	*CYP24A1*	20q13.2	rs2762934 (G > A)	0.50
			rs2762939 (G > C)	0.45
Cytochrome P450 3A4	*CYP3A4*	7q22.1	rs2740574 (C > T)	0.36
7-Dehydrocholesterol reductase	*DHCR7*	11q12.2	rs12785878 (G > T)	0.49
			rs3829251 (G > A)	0.41
Retinoid X receptor alpha	*RXRA*	9q34.2	rs7861779 (C > T)	0.48
			rs9409929 (G > A)	0.47
Vitamin D binding protein	*VDBP*	4q13.3	rs2070741 (T > G)	0.20
			rs7041 (A > C)	0.49
Vitamin D receptor	*VDR*	12q13.11	rs4516035 (*A1012G*, T > C)	0.48
			rs7975232 (*ApaI*, C > A)	0.50
			rs1544410 (*BsmI*, C > T)	0.48
			rs11568820 (*Cdx2*, C > T)	0.46
			rs731236 (*TaqI*, A > G)	0.47
			rs2238136 (C > T)	0.30
			rs7136534 (C > T)	0.41

*Note.* *MAF* highest minor allele frequency observed in any population including 1000 Genomes Phase 3, ESP and gnomAD (according to www.ensembl.org, last accessed on 2024-12-28); *SNP* single nucleotide polymorphism

### Vitamin D status reference values

In this study, serum 25(OH)D levels were measured in ng/mL and converted to nmol/L using the formula 1 ng/mL = 2.5 nmol/L (National Institutes of Health 2023). In accordance with previously published guidelines addressing the pleiotropic effects of vitamin D, we categorized serum 25-hydroxyvitamin D [25(OH)D] levels as ‘deficient’ (< 20 ng/mL, < 50 nmol/L), ‘insufficient’ (20–30 ng/mL, 50–75 nmol/L), and ‘sufficient’ (≥ 30 ng/mL, ≥ 75 nmol/L) (Holick et al. 2011; Pludowski et al. 2018).

### Nutrition protocol

The quality and quantity of nutrition were assessed using a three-day nutrition protocol, analyzed with DGExpert (version v. 2.0.37), a software program developed by the German Nutrition Society. Athletes were instructed to document all consumed food, liquids, and supplements, including their respective quantities. The protocol specifically included a question about the use of supplements to ensure comprehensive reporting. Athletes were thoroughly briefed on the importance of accurate documentation (Kwan et al. 2010). Each protocol underwent a quality check before analysis, and a Physical Activity Value of 2 was used to account for the higher energy demands of elite athletes. Athletes were encouraged to submit their protocols within two weeks of their initial blood measurement. If multiple protocols were submitted, only the first was included in the analysis presented here.

### Handgrip strength

Grip strength was measured unimanually using a hand-held dynamometer (MicroFET2; Hoggan Scientific, Salt Lake City, USA). Participants sat on a chair at a table, with their positions adjusted so that both feet were on the floor, knees at a 90° angle, and forearms on the table with elbows flexed at 90°. The wrist joint of the measured side was extended while the other arm rested loosely on the table. Participants were instructed to begin the measurement with light pressure, then gradually increase their force during a 3-s countdown, aiming for maximum force at 0. They were then asked to continue pressing maximally for another 5–6 s until the test administrator called a stop. The test administrator provided verbal encouragement. Before the measurement, participants completed a practice trial and after that had two actual trials for each side. A third trial was conducted if participants failed to perform the measurement correctly. The maximum strength value in Newtons for each side was recorded. Relative strength values were calculated by dividing the absolute strength value by the athletes’ body weight (N/kg).

### Statistical analysis

All analyses were conducted using R (version 4.4.1, R Core Team, R Foundation for Statistical Computing, Vienna, Austria) and RStudio (version 2024.4.2.764, Posit team, Posit Software, PBC, Boston, MA, USA). Descriptive statistics are presented as mean ± standard deviation (SD). Correlation analysis was conducted using Spearman’s rho. To explore potential differences in serum 25(OH)D levels related to age group, sex, time of measurement, and discipline (indoor vs. outdoor disciplines), we implemented the Welch two-sample *t* test. To acknowledge the lack of appropriate UVB radiation for dermal synthesis of vitamin D in the months from October to March in Germany (Webb and Holick 1988; Wilson‐Barnes et al. 2020), we divided the measurement time into ‘summer’ (April–September) and ‘winter’ (October–March) periods. Regarding the categorization of disciplines, we primarily based our decision on their respective main place of competition. Hence, artistic gymnastics, ice hockey, rhythmic gymnastics, table tennis, trampoline gymnastics, and volleyball were categorized as indoor disciplines, while 3 × 3 basketball, bobsleigh, modern pentathlon, and skeleton were categorized as outdoor disciplines. Each SNP adherence to the Hardy–Weinberg equilibrium (HWE) was tested with the R package ‘HardyWeinberg’ (version 1.7.5) (Graffelman 2015) using an exact test. Multiple linear regression with tenfold cross-validation was used for each SNP to analyze genetic influences on serum 25(OH)D levels. The normality of the residuals was assessed via Q-Q-Plots. The assumption of homoscedasticity was tested using the Breusch–Pagan test and visual inspection of the residuals. If the homoscedasticity assumption is violated, heteroscedasticity consistent (HC) standard errors of type HC3 were calculated. If additional non-normal errors were evident, HC4 was implemented following Hayes and Cai (Hayes and Cai 2007) using the R package ‘lmtest’ (version 0.9–40) (Zeileis and Hothorn 2002). Multicollinearity and influential factors were analyzed with variance inflation factors (VIF) and Cook’s distance using the R package ‘car’ (version 3.1–2) (Fox and Weisberg 2019). The relationship between serum 25(OH)D levels and grip strength was examined using linear regression analysis. Therefore, the average grip strength of both hands was calculated. The level of statistical significance was set at *p* < 0.05. Given the multiple comparisons in this study, we used the Benjamini–Hochberg procedure to control the false discovery rate (FDR) for the four Welch two-sample *t* tests on vitamin d levels and the *p* values of the respective SNP genotypes. Figures were created using ‘ggplot2’ (version 3.5.1) (Wickham 2016).

## Results

### Vitamin D status

The mean serum 25(OH)D level in this population of elite German athletes was 30.98 ± 13.43 ng/mL (77.45 ± 33.58 nmol/L). Of the 474 included athletes (231 female), 39.5% (*n* = 187) had insufficient levels between 20 and 30 ng/mL (50–75 nmol/L), and 16% (*n* = 76) had serum 25(OH)D levels beneath 20 ng/mL (50 nmol/L). In total, 55.5% (*n* = 263) of the athletes demonstrated serum 25(OH)D levels below 30 ng/mL (75 nmol/L, Table 3). Of the 76 athletes with a deficient status, 44 were female, 53 were under 18, 48 were measured in summer, and 61 participated in an indoor discipline. Of the 187 athletes with an insufficient status, 98 were female, 95 were under 18, 113 were measured in summer, and 141 participated in an indoor discipline. Of the 211 athletes with a sufficient status, 89 were female, 85 were under 18, 119 were measured in summer, and 138 participated in an indoor discipline.

**Table 3:** Descriptive statistics of serum 25-hydroxyvitamin D [25(OH)D] status in German elite athletes (*N* = 474)

Vitamin D status	*n*	%	Mean ± SD	Median	Range
Deficient (< 20 ng/mL; < 50 nmol/L)	76	16	15.2 ± 3.69	15.75	5.1–19.9
Insufficient (20–30 ng/mL; 50–75 nmol/L)	187	39.5	24.9 ± 2.78	25	20–29.9
Sufficient (≥ 30 ng/mL; ≥ 75 nmol/L)	211	44.5	42.1 ± 12.17	38.5	30–100

### Exploration of influencing factors

**Fig. 1:**
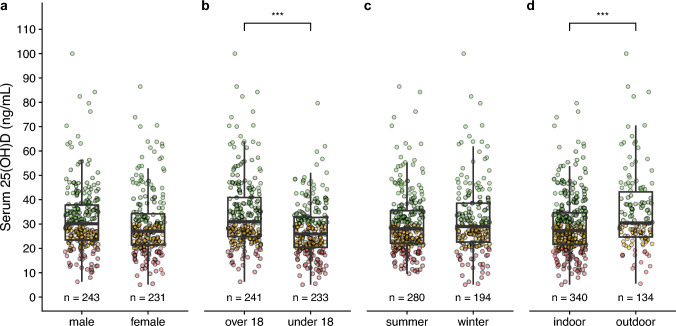
Serum 25-hydroxyvitamin D [25(OH)D] (ng/mL) compared by **a** sex, **b** age group, **c** season, and **d** indoor/outdoor discipline. The whiskers of each boxplot extend to ± 1.5 times interquartile range. Asterisks (***) indicate significant differences between groups (*p* < 0.001). Individual data points are colored based on serum 25(OH)D levels: red for levels below 20 ng/mL (50 nmol/L), yellow for levels between 20 ng/mL and 30 ng/mL (50–75 nmol/L), and green for levels above 30 ng/mL (75 nmol/L). The use of color is for illustrative purposes only and is not essential for understanding the figure

Regarding the effects of potential influencing factors, we examined serum 25(OH)D levels in relation to sex, age group (athletes under the age of 18 years vs. athletes over 18), season (summer vs. winter), and discipline (indoor vs. outdoor). Significant differences were observed for age group, with higher serum 25(OH)D levels in senior athletes compared to junior athletes (34.39 ± 14.76 ng/mL [85.98 ± 36.9 nmol/L] vs. 27.46 ± 10.84 ng/mL [68.65 ± 27.1 nmol/L]), t(440.59) = 5.84, *p* < 0.001, 95% CI [4.6, 9.27] (Fig. 1b). Similarly, athletes primarily competing in outdoor sports had higher serum 25(OH)D levels (35.15 ± 16.10 ng/mL [87.88 ± 40.25 nmol/L]) compared to those in indoor sports (29.34 ± 11.85 ng/mL [73.35 ± 29.63 nmol/L), t(192.36) = – 3.79, *p* < 0.001, 95% CI [ – 8.83, – 2.79] (Fig. 1b**/d**). The *p* values of age group and discipline were still < 0.001 after correcting for multiple tests.

No significant differences were found regarding sex, t(471.7) = 1.73, *p* = 0.085, 95% CI [ – 0.29, 4.55], and season, t(374.32) = – 1.15, *p* = 0.25, 95% CI [ – 4.02, 1.05] (Fig. 1a**/c**).

### Nutrition protocols

Overall, 226 nutrition protocols were evaluated. The estimated nutritional mean intake of vitamin D was 2.6 ± 2.55 µg/day. Considering the additional intake by supplementation, the combined mean intake of vitamin D was 10.8 ± 23.7 µg/day, ranging from 0.05 to 144 µg/day. Thirty-eight athletes (21 female) reported a supplementation of vitamin D. Of these 38 athletes, 14 athletes were under 18, and 22 competed in outdoor disciplines. Of the 226 athletes, only 34 met the German Nutrition Society’s recommended daily vitamin D intake of 20 µg through their diet and supplementation. Vitamin D supplementation ranged from 252 international units (IU)/day to 5762 IU/day. The combined intake (nutritional vitamin D + supplementation) differed between age groups, indicating higher values for athletes older than 18 (16.39 µg/day vs. 6.62 µg/day), *t*(136.84) = 2.89, *p* = 0.004, 95% CI [3.09, 16.45]. There was a small positive association between vitamin D intake (nutritional vitamin D + supplementation) and serum 25(OH)D concentrations (Spearman’s *r* = 0.19, *p* < 0.005).

### Analysis of vitamin D pathway related single nucleotide polymorphisms

All analyzed SNPs were in HWE (*p* > 0.05). Table 4 presents the mean ± SD of serum 25(OH)D levels in ng/mL concerning the analyzed SNPs, including their respective homozygous and heterozygous genotypes (e.g., AA, AC, or CC for *VDBP* rs7041). Age and indoor/outdoor discipline were included as control variables. Since observations of *CUBN* rs3740165 CC homozygous, *CYP3A4* rs2740574 CC homozygous, and *VDBP* rs2070741 GG homozygous were < 5, we merged these individuals with the respective heterozygous group as tenfold cross-validation could otherwise not be computed. It should be noted that for all SNPs except *VDBP* rs7041, we do not have genetic information regarding eight athletes. For *VDBP,* we do not have genetic information regarding nine athletes. These missing data are partly due to technical issues during the genotyping process, resulting in a no call for the associated alleles or that at the time of writing, the genotyping for these athletes was not yet finalized as the “in:prove” project is still ongoing.

**Table 4:** Serum 25-hydroxyvitamin D [25(OH)D] (ng/mL) concerning single nucleotide polymorphism (SNP) genotype. Data are mean ± SD

CUBN rs3740165	*CC*	*TC*	*TT*
*n*	1	41	424
Serum 25(OH)D	38	28.58 ± 12.28	31.21 ± 13.54
CYP24A1 rs2762934	*AA*	*AG*	*GG*
*n*	13	138	315
Serum 25(OH)D	31.88 ± 12.35	31.59 ± 13.68	30.7 ± 13.39
CYP24A1 rs2762939	*CC*	*GC*	*GG*
*n*	29	178	259
Serum 25(OH)D	27.79 ± 11.51	32.88 ± 15.48	30.06 ± 11.91
CYP3A4 rs2740574	*CC*	*TC*	*TT*
*n*	4	50	412
Serum 25(OH)D	17.28 ± 9.77	28.93 ± 12.05	31.38 ± 13.55
VDBP rs2070741	*GG*	*TG*	*TT*
*n*	*2*	*73*	*391*
Serum 25(OH)D	21.55 ± 1.06	32.87 ± 14.68	30.69 ± 13.19
VDBP rs7041*	*AA*	*AC*	*CC*
*n*	88	247	130
Serum 25(OH)D	25.43 ± 11.78	32.94 ± 13.33	31.04 ± 13.75
DHCR7 rs12785878	*GG*	*TG*	*TT*
*n*	45	194	227
Serum 25(OH)D	33.08 ± 13.23	29.23 ± 13.34	32.08 ± 13.42
DHCR7 rs3829251	*AA*	*AG*	*GG*
*n*	21	136	309
Serum 25(OH)D	31.53 ± 14.52	31.04 ± 14.09	30.94 ± 13.09
RXRA rs7861779	*CC*	*TC*	*TT*
*n*	325	124	17
Serum 25(OH)D	30.25 ± 13.07	33.3 ± 14.61	28.46 ± 8.92
RXRA rs9409929	*AA*	*AG*	*GG*
*n*	57	217	192
Serum 25(OH)D	33.04 ± 14.39	30.25 ± 12.38	31.22 ± 14.24
VDR rs4516035	*CC*	*TC*	*TT*
*n*	84	223	159
Serum 25(OH)D	32.11 ± 13.93	31.36 ± 13.33	29.89 ± 13.3
VDR rs7975232	*AA*	*AC*	*CC*
*n*	114	221	131
Serum 25(OH)D	29.9 ± 12.79	30.34 ± 12.64	33.05 ± 15.05
VDR rs1544410	*CC*	*TC*	*TT*
*n*	193	206	67
Serum 25(OH)D	31.82 ± 14	30.55 ± 12.87	29.96 ± 13.49
VDR rs11568820	*CC*	*TC*	*TT*
*n*	301	144	21
Serum 25(OH)D	31.27 ± 13.33	31.09 ± 13.69	26.39 ± 12.74
VDR rs731236	*AA*	*AG*	*GG*
*n*	204	197	65
Serum 25(OH)D	31.58 ± 13.99	30.62 ± 12.82	30.27 ± 13.57
VDR rs2238136	*CC*	*TC*	*TT*
*n*	242	190	34
Serum 25(OH)D	30.56 ± 12.83	30.77 ± 13.72	35.37 ± 15.46
VDR rs7136534	*CC*	*TC*	*TT*
*n*	270	173	23
Serum 25(OH)D	31.52 ± 13.49	30.42 ± 13.81	29.08 ± 9.23

*Note.* Serum 25(OH)D concentrations can be converted to nmol/L using the formula: 1 ng/mL = 2.5 nmol/L. SNPs with significant regression results are marked with an asterisk (*)

Regarding *Vitamin D binding protein* (*VDBP* rs7041) analysis of assumptions indicated deviation from both normality and homoscedasticity (Breusch–Pagan test = 18.60, *p* < 0.001). VIFs were below the threshold of 10. Cook’s distances were < 0.06 for all observations. Heteroscedasticity consistent (HC4) standard errors were used.

The model intercept was $\widehat{\upbeta }$ = 11.86 (*Standard error [SE]* = 3.59, *p* = 0.001). Both, the heterozygous AC genotype as well as the homozygous CC genotype were positively associated with serum 25(OH)D levels ($\widehat{\upbeta }$ = 7.46, *SE* = 1.59, *p* < 0.001 and $\widehat{\upbeta }$ = 6.23, *SE* = 1.81, *p* = 0.001, respectively). These results remained statistically significant after multiple testing correction. Further, the predictors age and ‘outdoor’ were positively associated with serum 25(OH)D ($\widehat{\upbeta }$ = 0.63, *SE* = 0.18, *p* = 0.001 and $\widehat{\upbeta }$ = 4.33, *SE* = 1.58, *p* = 0.007, respectively). Regarding the test data, MAE and RMSE averaged over all ten folds were 9.33 ± 0.74 ng/mL (23.33 ± 1.85 nmol/L) and 12.52 ± 1.66 ng/mL (31.3 ± 4.15 nmol/L), respectively. On average, the test models explained 14% (7% SD) of the variance in serum 25(OH)D (adjusted *R*^*2*^ = 0.06 ± 0.08).

After multiple testing correction of the respective genotypes, no other SNPs revealed associations with serum 25(OH)D levels. The predictors’ age and discipline were, on average, fundamental in all 17 models (for detailed results see **SI2**).

### Analysis of handgrip strength and vitamin D levels

Of all 474 athletes, 404 were examined regarding their handgrip strength. Analysis of assumptions indicated normality and homoscedasticity (Breusch–Pagan test = 0.08, *p* = 0.77). Cook’s distances were < 0.09. The model was significant (*F*(1, 402) = 17.82, *p* < 0.001), explaining 4% of the variance (adjusted *R*^*2*^ = 0.04).

**Fig. 2:**
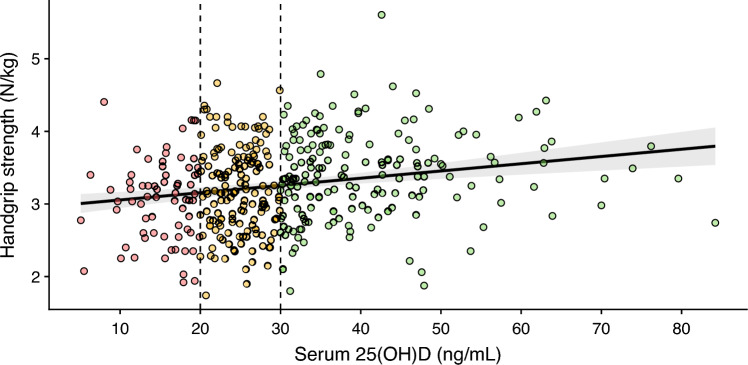
Association between handgrip strength and serum 25-hydroxyvitamin D [25(OH)D] (ng/mL) levels. The regression line is shown with the 95% confidence interval for the predictions. Dashed lines at 20 ng/mL (50 nmol/L) and 30 ng/mL (75 nmol/L) represent cut-off-values for deficient and insufficient vitamin D status, respectively. Individual data points are colored based on serum 25(OH)D levels: red for levels below 20 ng/mL (50 nmol/L), yellow for levels between 20 ng/mL and 30 ng/mL (50–75 nmol/L), and green for levels above 30 ng/mL (75 nmol/L). The use of color is for illustrative purposes only and is not essential for understanding the figure

The intercept had a $\widehat{\upbeta }$ = 2.96 and *p* < 0.001 (SE = 0.08, *t* = 38.24). Serum 25(OH)D levels were positively associated with handgrip strength ($\widehat{\upbeta }$ = 0.01, SE = 0.00, *t* = 4.22, *p* < 0.001). Specifically, for each 1 ng/mL (2.5 nmol/L) increase in serum 25(OH)D, handgrip strength was associated with an increase of 0.01 N/kg (Fig. 2).

## Discussion

In this cross-sectional analysis, we aimed to (1) assess serum 25(OH)D levels and the prevalence of insufficiency, (2) identify key factors influencing serum 25(OH)D levels, including genetic polymorphisms, seasonal variation (summer vs. winter), and discipline (indoor vs. outdoor), and (3) analyze the association between vitamin D levels and handgrip strength in German elite athletes.

### Prevalence of serum 25(OH)D insufficiency

Our study reveals a substantial prevalence of vitamin D insufficiency in German elite athletes, with 55.5% having deficient or insufficient serum 25(OH)D levels. In detail, we see a prevalence of vitamin D deficiency at 16%, which translates to every sixth athlete in the investigated population. Taking a closer look, the prevalence of athletes with a deficient or insufficient vitamin D status varies between junior (under 18) and senior (over 18) athletes, with 63.5% and 47.7%, respectively. These results are similar to the ones reported by former studies for the general German population (61.5%) (Rabenberg and Mensink 2016) and international elite athletes. Farrokhyar et al. reported a vitamin D insufficiency prevalence of 56% in elite athletes (the authors set the cutoff at 32 ng/mL [80 nmol/L]) (Farrokhyar et al. 2015).

### Factors influencing serum 25(OH)D levels

In a recent meta-analysis, Harju and colleagues reported a lower mean prevalence of vitamin D insufficiency of 30% in adults and 39% in adolescents (the authors set the cutoff at 20 ng/mL [50 nmol/L]) (Harju et al. 2022). In our population, senior athletes have higher mean serum 25(OH)D levels than junior athletes, who show mean serum 25(OH)D levels in the predefined insufficient range. This age effect is likewise present in the self-reported nutritional uptake of vitamin D (combined intake of diet and supplementation). This may partially be attributed to senior athletes’ longer involvement in professional sports and, subsequently, a higher level of awareness for a performance-aligned diet (Riviere et al. 2021). One consequence of this could certainly be a more intensive support for young athletes and early education about the importance of vitamin D. Further, more frequent vitamin D status checks could be considered.

Exposure to sunlight was also identified as an influencing factor. Following previous analyses (Farrokhyar et al. 2015), we see higher levels of 25(OH)D in athletes competing in outdoor disciplines compared to indoor disciplines. This is probably due to athletes of indoor disciplines training more often in sports centers and, therefore, having less UV exposure (Maruyama-Nagao et al. 2016). Interestingly, we did not see differences in serum 25(OH)D between measurements taken in the winter period (October–March) and the summer period (April–September). This effect of seasonal variation was repeatedly reported in previous studies, indicating higher levels in the summer period (Farrokhyar et al. 2015). The lack of seasonal patterns may be masked by different factors, such as diet and supplementation, the observed discipline (indoor/outdoor) effects, or the definition of the periods itself, with April and October potentially carrying over effects of the past season.

Exploring the genetic contribution of SNPs concerning serum 25(OH)D levels, we focused on 17 polymorphisms associated with the vitamin D pathway (Table 2) and evaluated their predictive qualities using multiple linear regression. We identified the gene *VDBP* rs7041 as a vital predictor of serum 25(OH)D. Before applying multiple testing corrections, *CYP3A4* rs2740574 and *RXRA* rs7861779 also demonstrated an association with serum 25(OH)D levels. These findings suggest that these genes may represent potential targets of interest for future research.

The *VDBP* is located on the human chromosome 4 (Bollen et al. 2023; Yang et al. 1985) and is responsible for the binding and transport of all vitamin D metabolites. *VDBP* is one of the most frequently studied genes regarding vitamin D status (Krasniqi et al. 2021). Among its variants, the rs7041 SNP involves an A > C substitution, leading to an amino acid change from aspartate to glutamate. This affects the binding activity of VDBP for 25(OH)D (Krasniqi et al. 2021), which in turn may alter serum 25(OH)D levels. In our study, the C allele was substantially associated with higher serum 25(OH)D levels in the investigated athletes. In detail, carriers of the homozygous CC and heterozygous AC genotype demonstrated higher levels compared to homozygous AA carriers. The regression model explained, on average, 14% of the variance in serum 25(OH)D, which is consistent with previous research (Manousaki et al. 2020; Revez et al. 2020) (for detailed results see **SI2)**. This suggests that the rs7041 SNP may serve as a genetic marker for identifying individuals at risk of lower serum 25(OH)D levels. These results are supported by prior studies such as those of Karcıoğlu Batur and colleagues, which demonstrated higher serum 25(OH)D levels following vitamin D supplementation in individuals with the CC genotype compared to AC and AA genotypes (Karcıoğlu Batur et al. 2021). Furthermore, the systematic review by Krasniqi et al. corroborates the relevance of rs7041, with approximately 69% of included studies confirming its association with vitamin D status. Despite these promising results, the review concluded that this (and other vitamin D related) SNP requires further validation to warrant its use as a genetic marker for identifying individuals at risk of vitamin D insufficiency (Krasniqi et al. 2021). Our findings further validate the current evidence on this specific SNP and highlight its potential role in individualized nutrition and supplementation strategies. However, to fully establish its practical utility, further studies are needed to evaluate its effectiveness as part of targeted interventions aimed at optimizing vitamin D status.

### The association of serum 25(OH)D and handgrip strength

Finally, our results show a significant positive association between handgrip strength and vitamin D levels, suggesting that adequate vitamin D levels are linked to better muscle function. However, it is important to note that this is an association and does not imply causation. Furthermore, the evidence regarding the relationship between muscular strength, especially grip strength, and exercise performance is currently inconclusive (Kim et al. 2020; Seo et al. 2019; Weiss et al. 2022; Wilson-Barnes et al. 2021; Yoon et al. 2021). For example, Książek and colleagues found a similar correlation (*r* = 0.22) between grip strength and serum 25(OH)D levels in Polish elite judoists (Książek et al. 2018), nonetheless the same authors found no association in Polish professional football players (Książek et al. 2016). Wium Geiker et al. found higher grip strength in male swimmers with sufficient vitamin D status compared to those with insufficient (< 20 ng/mL; < 50 nmol/L) vitamin D status (Wium Geiker et al. 2017). Among other factors, a clear association may be masked by the high heterogeneity between studies in terms of the studied populations, geographical location, discipline or the already highly developed level of muscular strength especially in elite athletic populations. Nonetheless, sufficient vitamin D levels are vital to athletes as low vitamin D levels have been associated with poor bone health and reduced muscle and immune functions, leading to a low muscle regenerative capacity and a higher risk for upper respiratory tract infections (Bergman et al. 2013; Dahlquist et al. 2015; Montenegro et al. 2019). Most and colleagues showed that athletes with serum 25(OH)D levels ≥ 30 ng/mL (≥ 75 nmol/L) achieved a higher maximum workload and maximal aerobic power compared to athletes with levels < 30 ng/mL (< 75 nmol/L) (Most et al. 2021). The underlying mechanisms of vitamin D on skeletal muscle emerge to be both direct and indirect via alterations in calcium and phosphate homeostasis and the VDR, respectively (Ceglia and Harris 2013; Dzik and Kaczor 2019). For example, Endo et al. showed smaller muscle fiber size in mice with non-functional VDRs compared to wild-type mice (Endo et al. 2004).

### Limitations

This study has some limitations that need to be addressed. First, the assessment of vitamin D status relied on a single blood sample. While this provides a snapshot of the athletes’ vitamin D levels, it does not account for temporal fluctuations, influenced by factors such as sun exposure, skin type or pigmentation, clothing style, use of sunscreen, diet, and supplementation. However, it was not always possible to record aforementioned factors in a standardized and controlled manner. Although our analysis accounted for seasonal variability, the cross-sectional design may not fully capture the dynamic nature of vitamin D levels throughout the year.

Dietary intake and supplementation were recorded using a three-day nutrition protocol, which depend on the athletes’ accurate and complete documentation. Despite briefing athletes on the importance of thorough reporting, some protocols were not completed as comprehensively as intended, introducing potential reporting bias.

### Conclusions and future directions

This study emphasizes the significant prevalence of 55.5% for deficient or insufficient vitamin D levels among German elite athletes. These findings and the observed positive association between serum 25(OH)D levels and handgrip strength emphasize the need for targeted interventions to address vitamin D deficiency and insufficiency, particularly in younger athletes, who exhibit a higher prevalence of insufficiency than their older counterparts. The analysis revealed that age and discipline (indoor vs. outdoor) significantly influence vitamin D status, with older elite athletes and outdoor athletes having higher serum 25(OH)D levels. These findings suggest that prolonged involvement in professional sports could improve awareness and practices related to vitamin D sufficiency.

Given the high prevalence of insufficient vitamin D levels, future research should focus on developing comprehensive strategies to reduce this risk in elite athletes. These strategies could include nutritional education and supplementation programs that emphasize the importance of vitamin D for both athletic performance and overall health. Tailored supplementation plans should be emphasized for younger athletes and those participating in indoor sports, who may be at higher risk of insufficiency.

Furthermore, regular, periodic measurements of serum 25(OH)D levels are advocated. Ideally, such measurements should be scheduled at multiple timepoints across different seasons to account for temporal variation in vitamin D levels. Measuring all athletes at a specific timepoint, such as during the winter season when insufficiency risk is higher, could also provide valuable insights into vitamin D prevalence within this population. Further, future studies could benefit from a longitudinal design to provide a more comprehensive understanding of the influence of training periodization on dietary intake and supplementation.

Additionally, the timing and frequency of these measurements could be individualized based on the risk factors identified in this study, such as the *VDBP* rs7041 SNP, age, and participation in indoor sports. This approach would facilitate the implementation of personalized nutrition and supplementation strategies tailored to the unique needs of each athlete.

By addressing these areas, future initiatives can improve the vitamin D status of elite athletes, potentially enhancing their performance and overall health. This approach will also contribute to a better understanding of the complex interactions between genetics, lifestyle and nutrient status in this unique population.

## Supplementary Information

Below is the link to the electronic supplementary material.

Supplementary file1 (DOCX 21 KB) SI1 Participant flowchart

Supplementary file2 (XLSX 104 KB) SI2 Individual fold and average regression results for all analyzed SNPs

## Data Availability

Data described in the manuscript and analytic code will be made available upon request pending (application and approval).

## Abbreviations

1,25(OH)2D: 1,25-Hydroxycholecalciferol
25(OH)D: 25-Hydroxyvitamin D
CUBN: Cubilin
CYP24A1: Cytochrome P450 24A1
CYP3A4: Cytochrome P450 3A4
DHCR7: 7-Dehydrocholesterol reductase
GWAS: Genome-wide Association Studies
IU: International units
IOM: Institute of Medicine
LCMS: Liquid chromatography-mass spectrometry
RXRA: Retinoid X receptor alpha
SNP: Single nucleotide polymorphism
VDBP: Vitamin D binding protein
VDR: Vitamin D receptor
