# Correction: Vitamin D status and its determinants in German elite athletes

**DOI:** 10.1007/s00421-025-05965-1

**Published:** 2025-09-29

**Authors:** Sebastian Hacker, Claudia Lenz, Lukas Reichert, Robert Ringseis, Karen Zentgraf, Karsten Krüger

**Affiliations:** 1 Department of Exercise Physiology and Sports Therapy, Institute of Sports Science, Justus Liebig University Giessen, Kugelberg 62, 35394 Giessen, Germany; 2 Work Unit Movement and Exercise Science in Sports, Institute of Sport Sciences, Goethe University Frankfurt, Ginnheimer Landstraße 39, 60487 Frankfurt am Main, Germany; 3 Institute of Animal Nutrition and Nutrition Physiology, Justus Liebig University Giessen, Heinrich-Buff-Ring 26-32, 35392 Giessen, Germany

**Correction: European Journal of Applied Physiology (2024) 125:1549–1561** 10.1007/s00421-024-05699-6

In the original version of this article, there were numerical inaccuracies in the article. While these do not alter the main conclusions, they do affect the precision and accuracy of the reported results.

In the abstract, second sentence of the ‘Results’ section, which previously read as

After correction for multiple testing, significant influences on 25(OH)D levels were observed for the C allele of *VDBP* rs7041 (AC Genotype: $\widehat{\upbeta }$ = 7.46, *p* < .001; CC Genotype: $\widehat{\upbeta }$ = 6.23, *p* = .001).

but should have read

After correction for multiple testing, significant influences on 25(OH)D levels were observed for the C allele of *VDBP* rs7041 (AC Genotype: $\widehat{\upbeta }$ = 7.45, *p* < .001; CC Genotype: $\widehat{\upbeta }$ = 6.23, *p* = .005).

In the Results section, first, second, third and fourth sentence of the fourth paragraph under the section ‘Analysis of vitamin D pathway related single nucleotide polymorphisms’, which previously read as

The model intercept was $\widehat{\upbeta }$ = 11.86 (*Standard error [SE]* = 3.59, *p* = 0.001). Both, the heterozygous AC genotype as well as the homozygous CC genotype were positively associated with serum 25(OH)D levels ($\widehat{\upbeta }$ = 7.46, *SE* = 1.59, *p* < 0.001 and $\widehat{\upbeta }$ = 6.23, *SE* = 1.81, *p* = 0.001, respectively). These results remained statistically significant after multiple testing correction. Further, the predictors age and ‘outdoor’ were positively associated with serum 25(OH)D ($\widehat{\upbeta }$ = 0.63, *SE* = 0.18, *p* = 0.001 and $\widehat{\upbeta }$ = 4.33, *SE* = 1.58, *p* = 0.007, respectively).

but should have read

The model intercept was $\widehat{\upbeta }$ = 11.89 (*Standard error [SE]* = 3.4, *p* < 0.001. Both, the heterozygous AC genotype as well as the homozygous CC genotype were positively associated with serum 25(OH)D levels ($\widehat{\upbeta }$ = 7.45, *SE* = 1.51, *p* < 0.001 and $\widehat{\upbeta }$ = 6.23, *SE* = 1.72, *p* < 0.001, respectively). These results remained statistically significant after multiple testing correction (AC genotype adjusted *p* < 0.001, CC genotype adjusted *p* = 0.005). Further the predictors age and ‘outdoor’ were positively associated with serum 25(OH)D ($\widehat{\upbeta }$ = 0.63, *SE* = 0.17, *p* < 0.001 and $\widehat{\upbeta }$ = 4.33, *SE* = 1.49, *p* = 0.004, respectively).

Second sentence of the fifth paragraph under the section ‘Analysis of vitamin D pathway related single nucleotide polymorphisms’, which previously read as

The predictors’ age and discipline were, on average, fundamental in all 17 models (for detailed results see **SI2**).

but should have read

The predictors age and discipline were fundamental in all 17 models (for detailed results see **SI2**).

In Supplementary Information 2, due to an error in the statistical analysis the regression values under “average regression results” for each SNP require minor adjustments, similar to those made for *VDBP* rs7041 above. Additionally, the term “average regression results” may be misleading. Supplementary Information 2 has now been replaced with the correct file.

## Supplementary Information

Below is the link to the electronic supplementary material.

Supplementary file1 (XLSX 106 KB)

