# Supplementary material for: Vitamin D status and its determinants in German elite athletes
Source: Eur J Appl Physiol. 2025 Jan 4;125(6):1549–61. doi: 10.1007/s00421-024-05699-6 (PMC12174178; doi:10.1007/s00421-024-05699-6)
Supplement: Supplementary file 1 — Supplementary file1 (DOCX 21 KB) SI1 Participant flowchart [file 421_2024_5699_MOESM1_ESM.docx]

**Participant flowchart**
